# Phenotypic effect of mutations in evolving populations of RNA molecules

**DOI:** 10.1186/1471-2148-10-46

**Published:** 2010-02-17

**Authors:** Michael Stich, Ester Lázaro, Susanna C Manrubia

**Affiliations:** 1 Centro de Astrobiología (INTA-CSIC), Carretera de Ajalvir km 4, 28850 Torrejón de Ardoz, Madrid, Spain

## Abstract

**Background:**

The secondary structure of folded RNA sequences is a good model to map phenotype onto genotype, as represented by the RNA sequence. Computational studies of the evolution of ensembles of RNA molecules towards target secondary structures yield valuable clues to the mechanisms behind adaptation of complex populations. The relationship between the space of sequences and structures, the organization of RNA ensembles at mutation-selection equilibrium, the time of adaptation as a function of the population parameters, the presence of collective effects in quasispecies, or the optimal mutation rates to promote adaptation all are issues that can be explored within this framework.

**Results:**

We investigate the effect of microscopic mutations on the phenotype of RNA molecules during their *in silico *evolution and adaptation. We calculate the distribution of the effects of mutations on fitness, the relative fractions of beneficial and deleterious mutations and the corresponding selection coefficients for populations evolving under different mutation rates. Three different situations are explored: the mutation-selection equilibrium (optimized population) in three different fitness landscapes, the dynamics during adaptation towards a goal structure (adapting population), and the behavior under periodic population bottlenecks (perturbed population).

**Conclusions:**

The ratio between the number of beneficial and deleterious mutations experienced by a population of RNA sequences increases with the value of the mutation rate *μ *at which evolution proceeds. In contrast, the selective value of mutations remains almost constant, independent of *μ*, indicating that adaptation occurs through an increase in the amount of beneficial mutations, with little variations in the average effect they have on fitness. Statistical analyses of the distribution of fitness effects reveal that small effects, either beneficial or deleterious, are well described by a Pareto distribution. These results are robust under changes in the fitness landscape, remarkably when, in addition to selecting a target secondary structure, specific subsequences or low-energy folds are required. A population perturbed by bottlenecks behaves similarly to an adapting population, struggling to return to the optimized state. Whether it can survive in the long run or whether it goes extinct depends critically on the length of the time interval between bottlenecks.

## Background

The fate of evolving populations is determined by a number of intrinsic properties of the ensemble and extrinsic mechanisms that interact in a highly non-trivial manner. The natural mutation rate of populations and their size, the effect that such mutations have on fitness and how these effects vary with the state of the population, or the environmental perturbations they have to cope with, all are relevant variables conditioning long-term survivability [1].

Populations evolving in constant environments eventually reach a fitness plateau, often interpreted as an optimum in the fitness landscape. The time required to reach the optimum and the structure of the population at the mutation-selection equilibrium corresponding to that environment depend, in addition to the factors listed above, on the environment and on the initial state of the population. During adaptation, the effect of mutations is different from their effect at equilibrium, probably due, among others, to differences in the fitness of populations and to changes in the genomic context [2,3]. Though it is common to observe an increase of fitness during adaptation, situations where fitness decreases before the new plateau is attained are not rare [4-6]. Based on several experimental observations and in mathematical models separating genotype and phenotype, it seems that the rate of incorporation of new mutations in adapting populations proceeds regularly, while the phenotype experiences discontinuous changes [7,8]. This search-and-fix behaviour has characteristic time scales dependent on the mutation rate and the size of the population, such that efficient optimization will only be observed in environments that remain constant for a time span longer than that required to find and fix beneficial changes [9]. Often, the process of smooth adaptation in constant environments is systematically interrupted by external perturbations. Common disturbances are unavoidable environmental fluctuations or durable changes that constitute a new, previously inexperienced selection pressure. An extreme perturbation is represented by population bottlenecks, where one or few individuals face the task of reconstructing, usually in relatively short time, the whole population.

The relationship between the mutation rate of a population and the variability of the environment where it evolves has been a matter of interest for a long time [10-12]. In strictly constant environments there should be selection against mutability, while periodically varying environments would demand different types in the population (optimal at one or another period), such that mutation rates that minimize the genetic load of the population would be favoured [10]. Early theories assumed fitness values monotonically decreasing with the number of mutations accumulated by an individual [13], and recognised the effect that epistatic interactions could play. Subsequent studies focused on the distribution of beneficial effects [14] and, under the strong assumption that the mutational neighbourhood of a genome is random, predicted an exponential distribution of the differences between the parental and mutated phenotypes [15]. More recent studies have identified the relevant effect of fitness-dependent mutation rates [16] and the dependence of the effect of mutations on the environment where they occur [17]. Still, an increasing number of empirical observations [6,18] indicate that existing theories are not of general applicability [19,20], and encourage additional efforts to quantify the effect of mutations on phenotype. Knowledge of the functional relationship between genomic mutation rates and phenotypic changes is essential to further develop phenomenological theories of evolution and adaptation. This is a main motivation behind the efforts currently devoted to obtain the distribution of fitness effects of mutations. The activity in the field acknowledges the profound difficulty in deriving such a relationship and points to its conceivable non-universality.

Simple, computational models of evolution explicitly separating genotype and phenotype might be of great aid in bridging the gap between phenomenological theories and experimental observations. The former are built on empirical observations which, as of yet, are far from complete. Actually, most experimental observations deal with a relatively small number of cases and model organisms, such that generalization is still a difficult enterprise [21]. Another problem presented by the experimental data is the small number of beneficial mutations that can be identified. Drift and clonal interference cause many beneficial mutations, especially when they have small effects, to be lost. Occasionally, some of these small effect mutations reach appreciable frequencies in the population, though they can often be incorrectly classified as neutral mutations. Another source of trouble concerns the selection coefficients of the mutations responsible for adaptation. Initial studies concluded that most evolutionary change was due to mutations with a small effect on fitness [22,23]. However, more recent evidences assign a prominent role to large effect mutations, especially at the first stages of adaptation or when the fitness of the wild type in the new environment is low [19,20,24-26]. Studies carried out with bacterial or viral populations focused on the analysis of the distributions of the fitness effects of beneficial mutations produced prior to selection [18,27,28]. Some of these studies report a good agreement with an exponential distribution, even when there are large variations in the fitness values of the wild type across environments [28]. On the other hand, other studies on adaptation to environments in which the wild type has low fitness reveal deviations from an exponential distribution due to the increase in the amount of beneficial mutations with large effect on fitness [27]. Finally, a study combining simulation and experimental data suggests that, regardless of the underlying distribution of mutations accessible to individuals, adaptation can be well described by a similar distribution of successful mutations with a simple form, peaked around a single value [29].

The computational study of random ensembles of RNA sequences folding into their minimum free energy secondary structure (a proxy for the phenotype) has permitted one to assess the role played by compensatory mutations [2]. More recently, analyses of the distribution of fitness effects on such ensembles have led to the conclusion that fitness values among similar genotypes are correlated [30]. Evolving populations of RNA sequences subject to point mutations and selection on structure have been successfully used as a suitable framework to explore several other aspects of evolution in heterogeneous populations [31]. However, these populations differ fundamentally from random sets of sequences.

Genomes within a population have to be highly correlated, since they share a phylogenetic history and have experienced similar selection pressures.

The aim of this contribution is to investigate the internal organization of optimized, adapting and perturbed populations of RNA molecules evolving under different genomic mutation rates, with the goal of quantifying the effect of mutations on fitness and establishing links between the state of the population and the effect of mutations. In the case of optimized populations, different fitness landscapes are used to probe the generality of our results. The model we use does not presuppose any underlying distribution of mutational effects. Mutations arise according to the established mutation rate and their effects are not selected *a priori*. They depend on the specific change that they produce in the secondary structure of the evolving molecules, in particular subsequences, or in the energy of the folded state. Our model also permits us to analyze the multiple mutations simultaneously present in a population, independently of whether they become fixed or not. Thus, mutations with highly deleterious effects can be pinpointed and those with a very small effect can be distinguished from neutral changes.

## Results and Discussion

### Evolutionary algorithm

#### Selection and mutation

**Figure 1:**
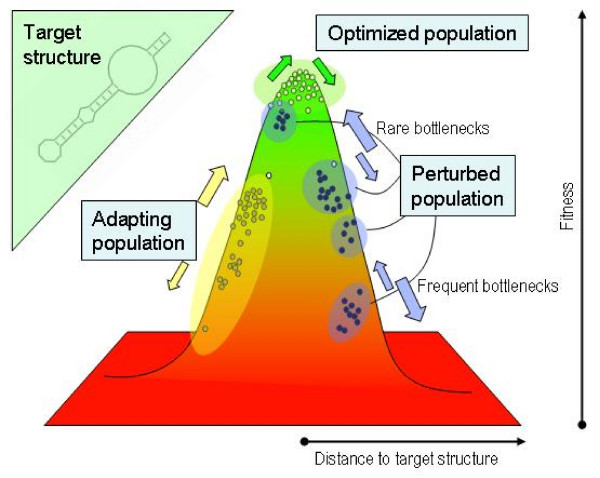
**Schematic representation of optimized, adapting, and perturbed populations in a simple fitness landscape**. The target structure *S *shown in the upper left corner represents the optimal adaptive solution. The distribution of phenotypes of an optimized population resides close to the optimum, where mutations with positive and negative effect on fitness (green arrows) compensate each other. An adapting population on its way to optimization is usually more dispersed in phenotype space, and beneficial mutations have a stronger effect than deleterious ones (yellow arrows). A population perturbed through severe bottlenecks is systematically displaced from equilibrium. Its ability to recover and survive in the long run depends on the frequency with which bottlenecks occur: for overly frequent bottlenecks, the population is pushed increasingly further from optimal states and eventually becomes extinct.

Our model system consists of a population of replicating RNA sequences, each of length *l *= 50 nucleotides (nt). We begin with an ensemble of *N *random sequences. The ensemble evolves through discrete generations and the population size is kept constant. An exception is the case of perturbations through population bottlenecks, to be discussed in detail below. At every generation, all the sequences in the population are folded into their minimum free energy secondary structure with help of the Vienna package (see Methods). We define a target secondary structure *S *(see Figure 1) towards which the evolution of the population is directed. The secondary structure of the molecules in the population is compared to *S*. The probability *p*(*d*_*i*_) that a molecule *i *in the population replicates is larger the closer to *S *it folds. This probability is defined as(1)

where *d*_*i *_is the distance between the structure corresponding to sequence *i *and the target structure *S*. As a measure of structural distance, we use the base-pair distance (see Definitions). The constant *l *is a scale factor, since the maximum base pair distance between two molecules of length *l *is proportional to *l*. The overall normalization factor is . The fitness function defined in Eq. (1) corresponds to the main fitness landscape to be studied in this work (*S*-landscape).

With the above definition of replication probability, the selective advantage *s *≡ [*p*(*d*_*i*_) - *p*(*d*_*j*_)]/*p*(*d*_*j*_) of a sequence *i *folding at distance *d*_*i *_to the target with respect to a sequence *j *folding at distance *d*_*j *_= *m *+ *d*_*i *_is independent of *d*_*i*_,(2)

This result implies that the selective advantage of a structure depends on the populational context, and not on its absolute distance to the target. Further, it reveals that the selection parameter *β *determines the relative advantage between different sequences in the population. In the limit *β *→ 0, *s *→ 0 for any two sequences: the distinction between molecules folding closer or farther from *S *is lost and all have asymptotically the same probability to replicate. When *β *→ ∞, only the sequence closest to the target is selected, the rest being eliminated in the next generation. The target structure *S *is found and fixed for most finite values of *β*, and only quantitative changes in the properties of the population are produced when it is varied. Increases in *β *can be compensated by decreases in *μ*, and *vice versa*. In this work, the value of this selection parameter will be fixed to *β *= 2.

When a molecule replicates, each nucleotide has a probability *μ *to be replaced by a nucleotide randomly chosen with equal probability among A, C, G, and U. In cases where the populations exceeds the maximum size *N*, we perform the usual Wright-Fisher sampling.

#### Definitions

The Hamming distance between two RNA sequences or subsequences is given by the number of positions in which their nucleotides differ. Structural differences can be estimated by various means. In this work, we apply the base-pair distance, given by the number of base pairs that have to be opened and closed to transform one structure into the other (as implemented in the RNAfold algorithm [32]).

A relevant macroscopic quantity to characterize the degree of optimization of the population is the fraction *ρ *of structures in the population folding into the target structure. This quantity is zero when our simulation starts: note that the probability that a random sequence of length *l *folds into an arbitrary secondary structure is negligibly small, of the order of 10^-15 ^in the present case [33] (but see also [34]). The maximum value of *ρ *is attained at the statistically stationary state, once the target structure is fixed in the population. The process of fixation and the dependence of *ρ *with the mutation rate *μ *have been described in detail elsewhere [9].

The change in fitness of an RNA molecule under replication with mutation rate *μ *is quantified by comparing the secondary structures of the mother *i *and daughter *j *sequences. We calculate the distances *d*_*i *_and *d*_*j*_, as described, and the difference Δ_*ij *_= *d*_*i *_- *d*_*j*_. If Δ_*ij *_= Δ > 0 (meaning that the mother sequence was farther from the target structure than its mutated daughter), the mutations increased fitness and count in the total of beneficial changes. If Δ_*ij *_= Δ < 0, mutations have caused a negative change in fitness, and the daughter sequence is farther from the target than her mother was. When Δ_*ij *_= 0, either mutations had no effect on fitness or no mutation has occurred.

This procedure is repeated over a large number of mother-daughter pairs (*i, j*), as we will specify, to obtain a probability distribution Π(Δ) of the changes in fitness. That distribution eventually yields the fraction of deleterious changes *p *and the fraction of beneficial changes *q*:(3)

The fraction of replication events with no change in fitness, *n' *= Π(0)-regardless of whether no mutations occurred or because the ones occurring were neutral-, can be immediately obtained from *p *and *q*: *n' *= 1 - *p *- *q*.

For a mutation rate *μ*, the probability that a sequence of length *l *acquires *k *mutations upon replication follows a Poisson distribution of average *μl*,(4)

The probability of incorporating no mutations is thus *P*(0) = *e*^-*μl*^, which for *μl *≪ 1 reduces to *P*(0) = 1 - *μl*. The fraction of truly neutral mutations is thus *n *= *n' *- *P*(0). If one wishes to calculate the fraction of deleterious, beneficial or neutral mutations conditional on at least one mutation having taken place, it suffices to divide the values of *p, q*, and *n *above by the expected number of sequences with one or more mutations, 1 - *P*(0). The effect of single mutations can be estimated in this way only for sufficiently small values of the mutation rate *μ*, since for relatively large values the probability of having two mutations in the same sequence becomes non-negligible. For example, for *μ *= 10^-3^, the probability that a sequence of length *l *= 50 is hit by two or more mutations is *P*(*k *≥ 2) = 1 - *P*(0) - *P*(1) ≃ 0.00121, thus one in a thousand molecules gets more than one mutation under replication. At sufficiently low mutation rates, the distribution of effects of mutations on fitness thus corresponds to the distribution of the effect of single mutations. As *μ *increases, sequences at a distance of two and more mutations from the parental molecule can appear in a single replication event. This has implications in the mobility of the population in sequence space and in its evolutionary dynamics [35].

The average selection coefficients *σ*_*q *_and *σ*_*p *_are calculated as the relative average change in fitness produced by all replication events affected by mutations with a beneficial or a deleterious effect on fitness, respectively:(5)

where *l *corresponds to the maximum fitness change, such that 0 < (*σ*_*p*_, *σ*_*q*_) < 1. The fraction of deleterious and beneficial mutations and the corresponding average selection coefficients are calculated while keeping a fixed value of the genomic mutation rate *μ*.

### Numerical results

In the following we investigate the response of populations of RNA sequences evolving under the situations described. The three different cases explored are schematically represented in Figure 1. The target structure selected in our simulations is depicted in the upper left corner. Starting with a random population of sequences, for values of the mutation rate below the error threshold *μ*_*c*_, the population is able to climb up towards the optimum of the phenotype space. After the transient, if mutation-selection equilibrium is reached, the population sits around the optimum, with a fraction *ρ *of correctly folding sequences determined by *μ*: the error rate at which a population evolves determines the degree of adaptation reached at the equilibrium. The mutation rate also determines the spread of the population in sequence and structure spaces. Populations perturbed through bottlenecks are forced to recover starting with a single sequence. If the time between bottlenecks is long enough, beneficial mutations can be found and fixed, recovery is possible, and the population is to be located near the optimum most of the time. However, for frequent perturbations it is likely that suboptimal sequences are repeatedly chosen. This favours the accumulation of deleterious mutations that separate the population steadily from the optimum: recovery is not possible, and eventual extinction might supervene.

**Figure 2:**
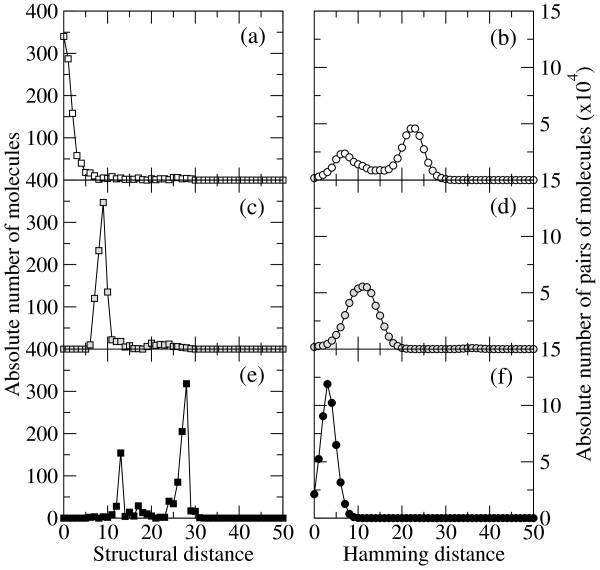
**Population diversity in phenotype and genotype spaces**. The phenotype of molecules is represented by their folded secondary structure. Phenotypes in an optimized population are close to the target structure and have typically a small structural distance to *S* (a). However, the population is spread in the space of genotypes (b). An adapting population is approaching the target (c). Due to the fast inheritance of the best (suboptimal) structure found, the dispersion in the space of genotypes is relatively small (d). A population subjected to periodic bottlenecks spreads in the space of phenotypes due to frequent perturbations (e). Since all molecules come from a recent parent sequence, the dispersion in the space of genotypes is small (f).

In the three cases described, and for a fixed value of the mutation rate, the diversity of each population in sequence and structure differs notably. Figure 2 compares the spread in phenotypes measured through the structural (base-pair) distance to the target *S* (plots (a), (c), and (e)) and the corresponding Hamming distance between all possible pairs of molecules in the population (plots (b), (d), and (f)). This quantitative picture agrees with the phenomenological description represented in Figure 1. The details of the numerical calculations are provided in the forthcoming subsections.

#### Optimized populations

We start the simulation as described, with *N *= 1000 random RNA sequences subject to selection as a function of their distance to the target structure *S*, Eq. (1). In the statistically stationary state, the population has attained its maximum degree of optimization and is located as close to the optimum (represented by the target structure) as allowed by the operating mutation rate *μ*. The fraction *ρ *of sequences folding into *S *is a decreasing function of *μ *for values of the mutation rate below the error threshold; *ρ *becomes strictly zero for *μ *> *μ*_*c *_only for sequences of infinite length. In our case, where sequences are short, there is a non-zero probability that the target structure is found, even above the error threshold. However, it is rapidly lost due to the high mutation rate. Hence, though we sporadically see the appearance of *S *above threshold, it cannot be fixed in the population.

The distributions shown in Figure 2(a) and 2(b) correspond to a mutation rate *μ *= 0.004, which yields a fraction *ρ *≃ 0.34 of correctly folded sequences. Larger values of *μ *would yield broader distributions and smaller values of *μ*, corresponding to larger values of *ρ*, would pack the population closer to the optimum. An important point is to notice that there is always place for improvement within the population for any *μ *> 0. Suboptimal sequences (with *d*_*i *_≥ 1) might incorporate mutations (mostly compensatory once at the mutation-selection equilibrium) that diminish their distance to *S *and thus increase their fitness, while optimal sequences (with *d*_*i *_= 0) might be affected by mutations that change their folded state, and thus count as deleterious. At equilibrium, the two processes balance so as to maintain the fraction *ρ *constant. The situation is different regarding the genomic diversity of the population. Though at the first generations after fixation of the target structure all sequences are similar, in successive iterations the population explores the neutral network of genotypes. This permits the population to expand in sequence space, displaying broad variability in sequences while maintaining the optimized phenotype. Further, the topological properties of the neutral network allow a deep exploration of the space of configurations, a feature that has been shown to underly the extreme plasticity observed not only in RNA sequence evolution *in silico *[7], but also in natural populations [8]. The reorganizations of the populations at the sequence level, which occur all the time, can be masked by the more visible process of adaptation, where the similarity between phenotypes and their degree of optimization is the dominant and obvious outcome of selection processes. The relationship between sequence and structure in RNA molecules illustrates the complex mechanisms relating the genomic level to its phenotypic expression, and constitutes a first, simple instance that allows us to measure the effect of mutations on fitness.

**Figure 3:**
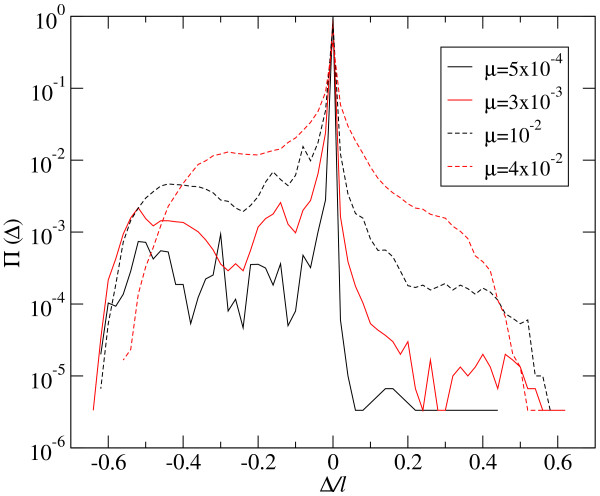
**Distribution of phenotypic changes - Optimized population**. The effect of mutations on the phenotype is quantified through the change in fitness caused upon replication. Four distributions corresponding to different values of the mutation rate *μ *are shown. The probability distribution Π(Δ) has been obtained by averaging over 300 generations in populations of size *N *= 1000. Its symmetry increases as *μ *grows, corresponding to populations that are more spread in the space of sequences and structures.

**Figure 4:**
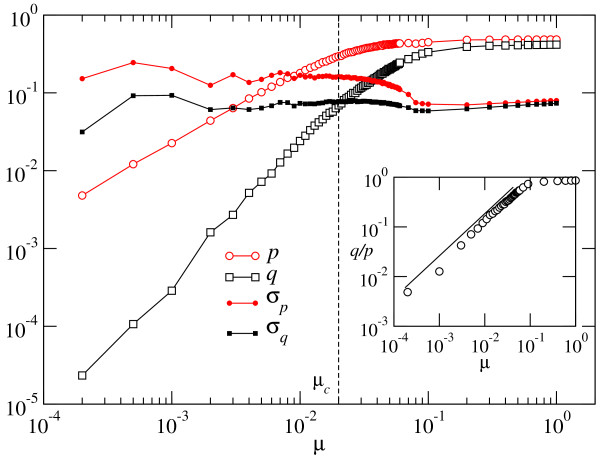
**Dependence of the phenotypic mutation fractions and of the average selective coefficients on the mutation rate *μ *- Optimized population**. The phenotypic mutation fractions *p *and *q *increase monotonically as *μ *grows. Below the error threshold they behave as a power-law of *μ*, to a good approximation. The average selective coefficients *σ*_*p *_and *σ*_*q *_show little variation with *μ*. The inset shows the ratio between the fraction of beneficial and deleterious mutations. The straight line serves as a guide to the eye.

At the mutation-selection equilibrium, the effect of deleterious mutations is compensated by the incorporation of compensatory mutations (counted as beneficial in the distribution of fitness effects), together with the replicative advantage of correctly folding sequences. Several examples of probability distributions Π(Δ) are shown in Figure 3. As can be seen, the total number of mutations of each sign depends on the mutation rate *μ*. Beneficial mutations are very rare at low values of *μ*, where the largest fractions *ρ *of correctly folded sequences occur. The fraction of mutations with deleterious effects is slightly larger, though it still has low absolute value. As could have been expected, most replication events do not change fitness, particularly because mutations are absent. As *μ *increases, mutations become more abundant, and changes of fitness upon replication increase in frequency. Simultaneously, the distribution becomes more symmetrical: the ratio between beneficial and deleterious mutations increases towards one. This analysis has been repeated for populations optimized at different values of *μ*. Figure 4 summarizes the obtained values of *p, q*, and the selection coefficients *σ*_*p *_and *σ*_*q *_as a function of the mutation rate *μ*. We have indicated the approximate position of the error threshold *μ*_*c*_. It is interesting to note that the effect of mutations on phenotype does not have any particular sensitivity to this important threshold. It only affects the probability of fixation of beneficial mutations, not their appearance, which increases monotonically with *μ*. This is consistent with previous observations of structural stability, a kind of collective effect in RNA populations, which still maintain a distributed signal of the target secondary structure despite the fact that no sequence in the population folds into it [9].

Below the critical error threshold, the phenotypic mutation fractions seem to bear a simple functional relationship with *μ*:(6)

with exponents α ≃ 0.89 ± 0.01 and γ ≃ 1.77 ± 0.02, indicating that, as the degree of adaptation of a population reduces as a consequence of evolution at progressively increased mutation rates, compensatory mutations increase their frequency at a higher speed than do deleterious mutations. We expect the algebraic relationship between phenotypic and microscopic mutation rates to be generic, although the specific values of the exponents α and γ will depend on the length of the sequences and on the particular secondary structure chosen. The ratio *q/p *quantifies the number of compensatory mutations per deleterious mutation. Its dependence with *μ *is displayed in the inset of Figure 4. Together with the functional relationships reported in Eq. (6), we obtain *q/p *≃ *μ*^ξ^, with *ξ *= *γ *- *α *= 0.87 ± 0.03.

The average selection coefficients vary only slightly with *μ*, with *σ*_*p *_taking about twice the value of *σ*_*q*_. This relationship is dependent on sequence length and target structure.

#### Adapting populations

The study of adapting populations is performed in a way similar to that for optimized populations. An ensemble of *N *= 1000 random RNA sequences is the starting point, and we replicate and select sequences as described. Now, however, fitness changes are measured before the population has found the target structure, such that it is formed by a population of phylogenetically related, suboptimal structures. In practice, we measure the structure of the population and the effect of mutations during 50 generations after the initial random state. The average number of generations required to find and fix the target structure is well above this value.

The internal structure of the population at generation 50 is illustrated through the representative example shown in Figure 2(c) and 2(d). The narrow peak around a distance *d *≃ 9 to the target structure in Figure 2(c) indicates that the phenotypes of the population are located close to a suboptimal structure at this distance. Note that most sequences are not folding into the best structure at this generation, since structures at distances smaller than the most abundant are also present. They will be likely fixed in subsequent generations, representing a further step towards optimization. Since the population is out of equilibrium and continuously jumping to increasingly better structures, the spread in the genome space is smaller than in the optimized case.

**Figure 5:**
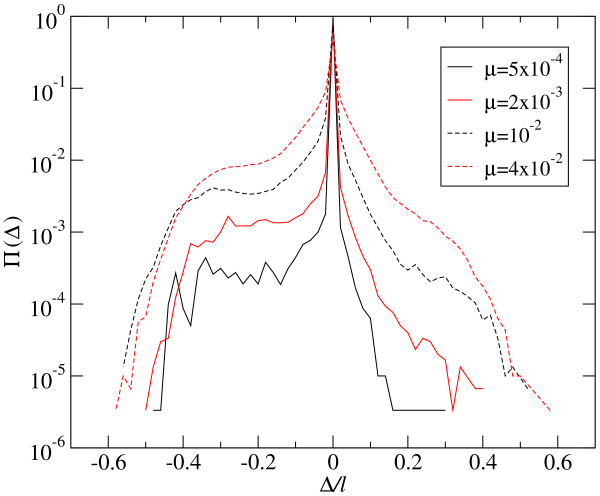
**Distribution of phenotypic changes - Adapting population**. Four distributions Π(Δ) corresponding to different values of the mutation rate *μ *for adapting populations are shown. The probability distribution has been obtained by averaging over the first 50 generations in six independent runs for populations of size *N *= 1000. Compare these results with those shown in Figure 3.

The distribution of changes in fitness along the first 50 generations of adaptation is shown in Figure 5 for four different values of the mutation rate *μ*. The overall shape of Π(Δ) is similar to that obtained at the mutation-selection equilibrium: a larger amount of deleterious mutations and a tendency of the distribution to become symmetrical as *μ *grows.

**Figure 6:**
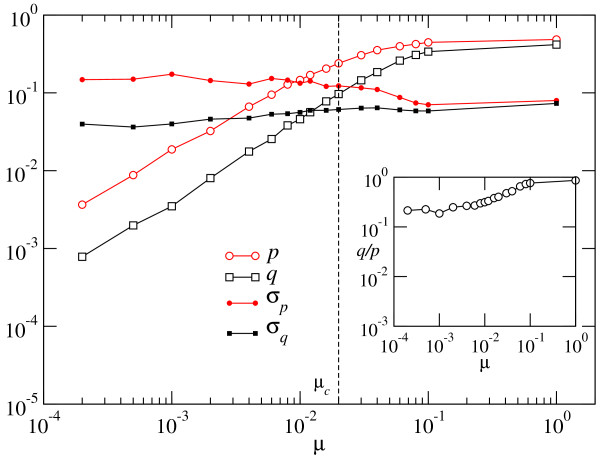
**Dependence of the phenotypic mutation fractions and of the average selective coefficients on the mutation rate *μ *- Adapting population**. Caption as in Figure 4. Compare the results shown in the two figures.

Figure 6 is the counterpart for adapting populations of Figure 4. It represents the average fraction of beneficial and deleterious mutations and the corresponding selection coefficients acting in the first 50 generations of evolution as a function of the mutation rate *μ*. In comparison to an optimized population, the fraction of deleterious mutations is slightly smaller than in the optimized case, while the fraction of mutations with a positive effect on fitness (whether they are truly beneficial or compensatory) is significantly larger. This fact is explained by considering that there are many more possibilities of improvement in the suboptimal states visited by an adapting population. This difference is bigger in the case of populations that evolve at low *μ *than in populations evolving at high *μ*. For *μ *values above the error threshold, adapting and optimized populations behave similarly. The functional dependence of the phenotypic mutation fractions with *μ *is of the same type as observed in an optimized population, Eq. (6). The exponents take different values in this case: using a least squares fit to the numerical data we obtain *α *= 0.93 ± 0.01 and *γ *= 1.06 ± 0.01. Note the remarkable quantitative difference in the behaviour of the ratio *q/p*, represented in the inset. Though still increasing as a function of *μ*, its variation with the mutation rate is much milder than in the case of optimized populations, as the corresponding exponent *ξ *= 0.13 ± 0.04 indicates. This result suggests that in the first stages of adaptation, populations evolving at different error rates are more similar than the same populations when equilibrium has been attained. In this case, however, substantial deviations observed in the *q/p *curve suggest that the power-law is not a good fit in the whole range of *μ*. The selective value of mutations is, both for beneficial and deleterious changes, typically lower than in the optimized case. Adaptation takes place through an increase in the fraction of beneficial mutations. In natural systems, mutations with a large effect on fitness can mask beneficial mutations with a small effect, which are then not identified during evolution (see [25] and references therein).

#### Perturbed populations

An optimized population can be shifted from equilibrium through the action of an external perturbation. Severe population bottlenecks, very common in the natural environment of highly heterogeneous populations such as RNA viruses, force the population to regenerate from one or a few individuals. The dynamics of this process can be simulated in our scenario. Consider an optimized population that has evolved until reaching mutation-selection equilibrium, as described. Take one of the sequences in the population and let it replicate during *g *generations. Now the growth of the population is unconstrained until reaching the size *N *= 1000.

In practice, the sequence selected at the bottleneck has an average number ⟨*c*⟩ of daughter sequences per generation defined as(7)

This dynamical rule applies now to the case of a growing population and substitutes the definition of replicative ability given in Eq. (1), the latter applying to populations of constant size. If the sequence at the bottleneck folds into the target structure, then ⟨*c*⟩ = 2, meaning that it duplicates on the average at each generation. Suboptimal sequences (with *d*_*i *_> 0) replicate at a slower pace. Note that the relative advantage of sequences folding into structures at relative distance *m *is the same as for the case of constant populations, Eq. (2).

If the number *g *of generations elapsed between bottlenecks is large enough, the population will be able to recover and again attain mutation-selection equilibrium. As *g *decreases, the time for optimization becomes shorter and, for *g *small enough, the total population will not be capable of achieving the optimum. Still, survival might be possible. At too low values of *g*, however, the perturbation becomes too strong and the few sequences after the bottleneck might be unable to replicate, in which case the population goes extinct.

For optimized and adapting populations, the mutation rate *μ *is the main source of randomness in the system, permitting as well change and improvement. Population bottlenecks constitute an additional source of stochasticity and represent a stronger perturbation, since selecting a random sequence to found a new population implies choosing a suboptimal structure with a probability of at least 1 - *ρ*. In those cases, the population faces two important difficulties: first, it has to go again through the adapting transient, where the target structure has not yet been found; second, the reduction in the population size impairs the capability of the ensemble to generate beneficial variants.

Figure 2(e) and 2(f) show the typical structure of a population 20 generations after a bottleneck. A sequence at a large distance from the optimum was selected by chance, such that, after 20 generations, the population is still far from finding the target structure, and formed at that moment by two groups that are identified as the two peaks in the distribution of structural distances. It is interesting how concentrated the population is in sequence space [Figure 2(f)], due to the clonal regeneration and the short time elapsed from the common ancestor of all sequences.

**Figure 7:**
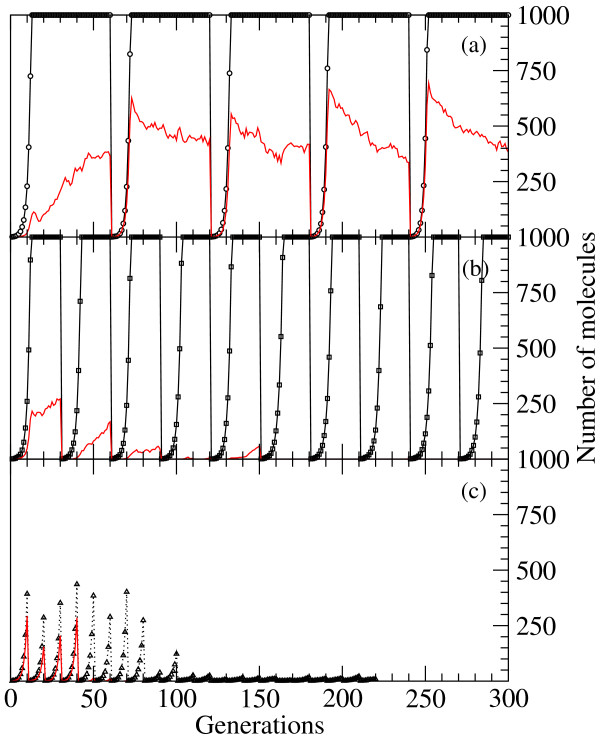
**Dynamics of a population subjected to regular bottlenecks**. The frequency of application of extreme population bottlenecks, where a single molecule has to regenerate the whole population, threatens the long-term survivability of the ensemble. We show three representative simulations corresponding to (a) 40; (b) 25; and (c) 10 generations between consecutive bottlenecks. Open symbols in black represent the total number of sequences in the population; red curves stand for the total number of correctly folded sequences. Note the variable number of generations required for saturation of the population size in plots (a) and (b).

The dynamics of three populations under the action of bottlenecks with different periodicity is illustrated in Figure 7. In Figure 7(a), a randomly chosen molecule of the population is selected every 40 generations to restart the growth process. For the parameters used, this interval is long enough to permit long-term survival. On the one hand, the total size of the population can always reach saturation. In most cases, even if a suboptimal structure was selected at the bottleneck, the system is able to find the target structure. According to our results, structures which are at a large distance from the target would experience a larger amount of beneficial mutations. Therefore, these molecules would benefit from increases in the error rate, an expectation for which there is experimental support. Viral clones extracted from an optimized population of the *Qβ *bacteriophage incorporated a lower amount of beneficial mutations than viruses isolated from a population previously displaced from mutation-selection equilibrium due to the application of successive bottlenecks [36].

In the possible case that an optimal sequence is selected, it replicates fast and reaches high density in a small number of generations. In this case, almost all sequences in the first generations after the bottleneck fold into optimal structures, thus generating a superoptimal, unstable population that slowly relaxes towards the diversity characteristic of the mutation-selection equilibrium under the action of the mutation rate. Figure 7(b) shows how an increase in the frequency of bottlenecks, here applied every 25 generations, represents a hazard to the population. We have seen extinction in a number of realizations with these parameters, while some other runs show survival for the whole length of the simulation. Extinction becomes systematic for bottlenecks applied too frequently. In Figure 7(c) the perturbation is applied every 10 generations and extinction is certain. Note that, irrespectively of the frequency of bottlenecks, any population suffering extreme bottlenecks is condemned to extinction, since there is a non-zero probability that the molecule chosen at the bottleneck fails to replicate in cases when it is far enough from the optimal structure. This argument applies to any number of molecules, though as the population increases in size this probability becomes negligibly small. The extension of these results to natural systems has to be done with due care. For example, extinction is rare in viral populations subject to bottlenecks even when these are applied at a frequency incompatible with the full regeneration of the population [37], due to the ability to recover fitness through a multiplicity of pathways.

The critical frequency of bottleneck application for the population to survive depends on other model parameters, especially on the mutation rate *μ*. Low values of *μ *correspond to a higher density *ρ *of correctly folded sequences, thus favouring selection of an optimal sequence at the bottleneck. However, a sufficiently low value of *μ *hinders the generation of diversity and with it the appearance of more optimal variants. Actually, when bottlenecks are frequent a good evolutionary strategy is to replicate under a not too low mutation rate, such that, in the case that a suboptimal phenotype is the founder of a new population, the time to recover mutation-selection diversity is minimized [38]. This represents an interesting compromise between time-scales: to guarantee long-term survivability, the number of generations between bottlenecks should not be smaller that the number of generations required to find and fix the target structure in the population.

#### Optimized populations in other fitness landscapes

An important question is whether our results are robust when different definitions of fitness for the RNA molecules are used. Up to now, we have only considered that, the closer a sequence folds to the target structure, the fitter it is. In this section, we introduce two additional definitions of fitness and discuss how they affect the dynamics and organization of optimized populations. As will be shown, the system is able to find a remarkably large number of genotypes compatible with the new selection pressures applied to the system, and the collective statistical properties of the population are only weakly affected.

Many molecules require a specific sequence of nucleotides for their function to be properly performed. Conserved subsequences often correspond to active sites which cannot be mutated, so variations in that subsequence are selected against. As an example, suppose that a target sequence *Q *of length *l*_*s *_nucleotides is required at a certain position for a sequence to have maximal fitness, and that deviations from that composition are penalized. A sensible way to add this requirement is the use of a fitness function of the form(8)

where  is the Hamming distance between the corresponding subsequence in molecule *i *and the target sequence *Q, l*_*s *_is the length of the target sequence, and *β*_*H *_is a selection parameter that measures the relative strength of selection for target sequence *Q *versus selection for target structure *S*. The landscape defined by Eq. (8) is the *S *+ *Q*-landscape. In the examples to be shown, we have chosen a target sequence GUAUCUUCAC, with *l*_*s *_= 10, placed at the 5' end of molecules in the population, and a selection parameter *β*_*H *_= 1. As previously, *β *= 2.

A second important property of folded molecules is their thermodynamical stability. Usually, sequences folding into configurations of lower energy are at an advantage with respect to those of higher energy. Suppose that there is an absolute minimum energy *E*_*m *_for sequences of length *l*, and call *E*_*i *_the energy of sequence *i *folded into its minimum free energy secondary structure. A suitable fitness function to favour low-energy configurations is(9)

where, analogous to previous definitions,  is the distance between the energy of molecule *i *and the reference energy *E*_*m*_, and *β*_*E *_is a selection parameter that again measures the relative strength of selection for low energy versus selection for target structure *S*. Equation (9) defines the *S *+ *E*-landscape. In the forthcoming examples, *E*_*m *_= -72 kcal/mol, which corresponds to the energy of a sequence of length *l *= 50 with 23 G=C pairs that contribute -3 kcal/mol each and where the positive contribution of the loop has been discarded. This value of *E*_*m *_is a lower bound to the energy of sequences of length *l *= 50. The selection parameter *β*_*E *_= 0.5, and *β *= 2. Let us compare the mutation-selection equilibria of three populations evolving on each of the landscapes described under a mutation rate *μ *= 0.003. In order to evaluate the degree of adaptation achieved for each of the traits under selection (structure, sequence, and energy), three relevant quantities describing the macroscopic state of these populations are used: as before, the density of sequences correctly folding into the target sequence, the average energy ⟨*E*⟩ = *N*^-1^∑_*i *_*E*_*i *_of the population, and the fraction *ρ*_*H *_of sequences at distance  = 0 from the target sequence *Q*. As previously performed, the simulation begins with an ensemble of *N *= 1000 random sequences. At each generation, the quantities *d_i_*,  and/or *E*_*i *_relevant in each landscape are evaluated. Substitution by a new generation of offspring sequences proceeds as described.

At the mutation-selection equilibrium, we observe the following. The population evolving in landscape *S *has *ρ *≃ 0.43, its average energy is ⟨*E*⟩ ≃ -12.3 kcal/mol and *ρ*_*H *_≃ 0. That is, the required secondary structure has been successfully found but essentially none of the subsequences matches *Q*. The energy of the population is comparable to that of an ensemble of randomly chosen sequences folding into the target structure *S*. When selection for sequence is turned on in the *S *+ *Q *landscape, there is a fraction *ρ *≃ 0.45 of sequences correctly folding into the target structure. However, this occurs simultaneously with a fraction *ρ*_*H *_≃ 0.85 of molecules bearing the correct target sequence *Q*. The average energy in this case is comparable to that in the previous landscape, ⟨*E*⟩ ≃ -13.0 kcal/mol. Hence, the population has been completely displaced from its position in the space of sequences (compared to landscape *S*), allowing the optimization of a second trait (target sequence) while keeping other macroscopic values essentially unchanged. The third population, evolving on landscape *S *+ *E*, is optimized at *ρ *≃ 0.36 with *ρ*_*H *_≃ 0. However, its average energy has significantly decreased to ⟨*E*⟩ ≃ -28.4 kcal/mol. This again speaks for a major displacement in sequence space with respect to the two previous landscapes, favouring those sequences folding into *S *with minimum energy, and with a composition that does not match the target sequence *Q*.

The target structure *S *and the target sequence *Q *can be simultaneously (and partly independently) optimized since *Q *is compatible with folding into *S*. This is not a general situation, since demanding a target sequence too long or too biased in its composition might prevent the existence of solutions fulfilling both requirements. This is also the case for the selection of *S *together with the requirement of a low folding energy. If the parameter *β*_*E *_would take an exceedingly high value, structures of low energy (different from *S*) would dominate the population. This is probably the reason why *ρ *takes a value slightly lower in landscape *S *+ *E *than in the two former landscapes. While it is important to take these possibilities into account, our results indicate that, in this model, the simultaneous optimization of two traits is possible for a broad range of selection parameters *β, β*_*H*_, and *β*_*E*_.

In addition to the different conceptual situations represented by the three landscapes introduced in this work, there are relevant geometrical differences among them. Selection for structure represents a rough landscape where changes in a single nucleotide can cause deep reorganizations in the folded configuration. On the contrary, selection for a specific sequence represents a smooth landscape where changes in the distance  are strictly proportional to the mutation rate. Actually, for a mutation rate *μ *the distribution of changes in  is a Poisson distribution of average *μl*, identical to Eq. (4). The landscape corresponding to selection for low energy shares characteristics with both. On the one hand, if mutations acquired through replication do not disrupt the folded state, changes in energy have to be small, since only the composition of the sequence is affected. However, if mutations lead to a different folded state, the minimum energy can suffer major changes, due to the appearance of a different distribution of structural motifs (stacks, loops and dangling ends) having a major role in the folded energy.

In the next section, we study the distribution of fitness effects Π(Δ^*L*^) corresponding to landscapes *S, S *+ *Q*, and *S *+ *E*. The superindex *L *is generic and corresponds to a different variable Δ^*L *^for each landscape. To make the three distributions quantitatively comparable, we have to use rescaled variables in the *x*-axes, analogous to *x *= Δ/*l*, as introduced for landscape *S*. They are *x*^*Q *^= Δ^*Q*^/*l *for landscape *S *+ *Q *and *x*^*E *^= Δ^*E*^/*l *for landscape *S *+ *E*, with(10)

where  is the change in distance to target sequence *Q *between a mother sequence *i *and its daughter sequence *j*, and(11)

with Δ_*E *_= *E*_*i *_- *E*_*j*_.

### The effects of mutations on fitness

In this section we discuss some of our results in quantitative detail. First, we perform a systematic analysis of the functional form of the distribution of fitness effects for optimized and adapting populations under selection for structure. Second, we analyse the effects on fitness for optimized populations in different fitness landscapes. Finally, we derive gross quantities that are usually measured in experimental research, as the fraction of mutations causing positive or negative changes in fitness conditional on the incorporation of a single mutation.

#### Statistical analysis of the distribution of fitness effects

We have used five different probability density functions to fit the numerical, cumulative distributions of the effects of mutations on fitness. Table 1 shows the functional form of the probability distributions (PD) assayed *P*(*x*) and of the corresponding cumulative probability function (CPF), . The results for optimized and adapting populations evolving under selection of the secondary structure are compiled in Table 2. Those parameters yield the best fit in each case, and have associated errors and *R*-squared values as shown. As a general trend, we observe that beneficial mutations typically have significantly smaller fitness effects than deleterious mutations - in agreement with the difference in the average selection coefficients calculated previously. This can be seen for instance in the *λ *parameter of exponential fits, in the consistent changes in the shape parameter *b *for Γ and *β *distributions, in the slope *a *of the Pareto distribution, or in the *σ *value of the Lognormal distribution.

**Table 1:** Functions used to fit numerical data

	Exponential[*λ*]	Γ**[*a, b*]**	*β*[*a, b*]	Pareto[*k, a*]	Lognormal[*m, σ*]
*P*(*x*)	*λe*^-*λx*^				

*Q*(*x *≤ Λ)	1 - *e*^-*λ*Λ^				

We have used five different probability distribution functions to fit the numerically obtained effects of mutations on fitness. We show the functional form of the probability distribution (PDF) *P*(*x*) and the corresponding cumulative probability distribution (CPF),  for each case. In our fits, we have used the latter, with parameters that yield the minimum least-squares deviation from our numerical results .

**Table 2:** Least squares fits to distributions of mutation effects on fitness

	Exponential[*λ*]	Γ**[*a, b*]**	*β*[*a, b*]	Pareto[*k, a*]	Lognormal[*m, σ*]
*μ *= 0.001	*λ *= 11.6 ± 0.6	*a *= 0.94 ± 0.14	*a *= 0.87 ± 0.14	*k *= 0.021 ± 0.001	*m *= 2.88 ± 0.05
*B*		*b *= 0.092 ± 0.015	*b *= 9.42 ± 1.70	*a *= 0.93 ± 0.03	*σ *= 1.03 ± 0.06
Optimized	*R*^2 ^= 0.940	*R*^2 ^= 0.940	*R*^2 ^= 0.934	*R*^2 ^= 0.982	*R*^2 ^= 0.969

*μ *= 0.004	*λ *= 11.4 ± 0.5	*a *= 1.32 ± 0.15	*a *= 1.21 ± 0.15	*k *= 0.022 ± 0.001	*m *= 2.79 ± 0.03
*B*		*b *= 0.065 ± 0.008	*b *= 13.2 ± 1.8	*a *= 0.95 ± 0.05	*σ *= 0.88 ± 0.03
Optimized	*R*^2 ^= 0.974	*R*^2 ^= 0.975	*R*^2 ^= 0.972	*R*^2 ^= 0.959	*R*^2 ^= 0.989

*μ *= 0.001	*λ *= 15.0 ± 1.0	*a *= 1.95 ± 0.32	*a *= 1.87 ± 0.32	*k *= 0.021 ± 0.001	*m *= 3.01 ± 0.04
*B*		*b *= 0.031 ± 0.006	*b *= 28.9 ± 5.2	*a *= 1.13 ± 0.04	*σ *= 0.76 ± 0.04
Adapting	*R*^2 ^= 0.950	*R*^2 ^= 0.969	*R*^2 ^= 0.967	*R*^2 ^= 0.985	*R*^2 ^= 0.984

*μ *= 0.004	*λ *= 13.4 ± 0.7	*a *= 1.83 ± 0.22	*a *= 1.73 ± 0.22	*k *= 0.021 ± 0.001	*m *= 2.89 ± 0.02
*B*		*b *= 0.039 ± 0.005	*b *= 23.0 ± 3.1	*a *= 1.06 ± 0.06	*σ *= 0.76 ± 0.03
Adapting	*R*^2 ^= 0.969	*R*^2 ^= 0.981	*R*^2 ^= 0.979	*R*^2 ^= 0.965	*R*^2 ^= 0.992

*μ *= 0.001	*λ *= 4.85 ± 0.16	*a *= 0.93 ± 0.09	*a *= 0.75 ± 0.06	*k *= 0.026 ± 0.003	*m *= 2.04 ± 0.05
*D*		*b *= 0.224 ± 0.024	*b *= 3.03 ± 0.27	*a *= 0.58 ± 0.05	*σ *= 1.12 ± 0.07
Optimized	*R*^2 ^= 0.962	*R*^2 ^= 0.964	*R*^2 ^= 0.972	*R*^2 ^= 0.840	*R*^2 ^= 0.947

*μ *= 0.004	*λ *= 6.15 ± 0.19	*a *= 1.27 ± 0.10	*a *= 1.06 ± 0.07	*k *= 0.026 ± 0.003	*m *= 2.17 ± 0.04
*D*		*b *= 0.126 ± 0.011	*b *= 5.70 ± 0.41	*a *= 0.68 ± 0.06	*σ *= 0.91 ± 0.05
Optimized	*R*^2 ^= 0.989	*R*^2 ^= 0.985	*R*^2 ^= 0.989	*R*^2 ^= 0.843	*R*^2 ^= 0.973

*μ *= 0.001	*λ *= 6.31 ± 0.18	*a *= 1.08 ± 0.09	*a *= 0.90 ± 0.07	*k *= 0.025 ± 0.003	*m *= 2.25 ± 0.04
*D*		*b *= 0.146 ± 0.014	*b *= 4.98 ± 0.42	*a *= 0.68 ± 0.05	*σ *= 1.00 ± 0.06
Adapting	*R*^2 ^= 0.982	*R*^2 ^= 0.980	*R*^2 ^= 0.982	*R*^2 ^= 0.868	*R*^2 ^= 0.970

*μ *= 0.004	*λ *= 7.86 ± 0.21	*a *= 1.19 ± 0.09	*a *= 1.03 ± 0.08	*k *= 0.024 ± 0.002	*m *= 2.44 ± 0.03
*D*		*b *= 0.106 ± 0.008	*b *= 7.28 ± 0.60	*a *= 0.78 ± 0.06	*σ *= 0.93 ± 0.04
Adapting	*R*^2 ^= 0.990	*R*^2 ^= 0.987	*R*^2 ^= 0.986	*R*^2 ^= 0.900	*R*^2 ^= 0.987

Representative examples of distributions of beneficial and deleterious fitness effects (*B *or *D *in the first column) for optimized and adapting populations and two different values of the mutation rate in each case. The parameters of the least-squares fits for five different (accumulated) probability distribution functions and the corresponding *R*-squared values are shown for each distribution.

**Figure 8:**
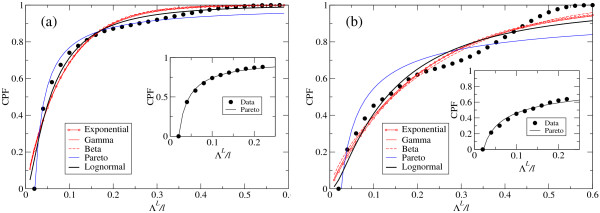
**Least-squares fits to the distribution of fitness effect**. (a) Cumulative distribution of beneficial effects of mutations in fitness. (b) Cumulative distribution of deleterious effects of mutations in fitness. In both cases, solid circles represent the numerical results of the simulations (see main text for details). Different functions fitting the data are shown, as specified in the legend. The insert shows the distribution of small effects (up to a 25% change in fitness), which is well fit by a Pareto distribution. The mutation rate is in both cases *μ *= 0.004.

If we were only to consider *R*-squared values corresponding to each fit as a measure of its goodness, we might conclude that most PDs represent the data reasonably well, though there is no function able to account for all numerical distributions. A closer inspection of the fits reveals systematic deviations from numerical results. Figure 8 depicts two representative examples of fits to (a) beneficial and (b) deleterious distributions of fitness effects in an optimized population. PDs dominated by an exponential decay (Exponential, Γ, and *β *distributions) underestimate the number of small effects and overestimate the number of average-to-large effects. In the case of the Γ and *β *distributions, the parameter *a *is in almost all cases close to 1 (a value of *a *= 1 corresponds to a pure exponential decay). This reveals that the fit to data is not improved by considering those two-parameter functions instead of the simpler, one parameter, exponential. The three functions are actually very similar. The Lognormal distribution slightly improves the fit in the region of small effects: It represents a best fit to beneficial distributions (which, as discussed, have more weight in small effects), though it systematically fails in the same regions as functions characterized by exponential decays.

None of the functions assayed are thus able to explain the shape of the distributions obtained in the whole domain of changes in fitness. One reason might be that our sequences are relatively short and thus fold into small secondary structures. We cannot discard that large effects on fitness could be affected by structural motifs of the particular target secondary structure studied in this work (e.g. the prominent shoulder observed in several distributions of deleterious fitness effects is probably caused by global disruptions of the target secondary structure).

The distribution of small effects on fitness, which considers mutations that slightly change the secondary structure of the parent sequence, should be much less affected by specific properties of the structure studied. We have thus conducted a systematic study of the distribution of small fitness effects, defined as those replication events affected by mutations where the change in the secondary structure was at most 22% with respect to the parent structure. This range is not too small: it can explain around 90% of the beneficial changes observed and around 70% of the deleterious changes (see Table 3). Least-squares fits to numerical data in that range yield a very good agreement between data and function: the *R*-squared coefficients are at least as good as the best fit in the whole range of fitness change, but now there are no systematic deviations from data. Two representative examples of Pareto distribution fits to numerical data are shown in the inserts of Figure 8(a) and 8(b).

**Table 3:** Least-squares fit of a Pareto function to the distribution of small effects on fitness

	Pareto[*k, a*]	% of mutations
*μ *= 0.001, *B *Optimized	*k *= 0.0202 ± 0.0003	88.1
	*a *= 0.848 ± 0.014	*R*^2 ^= 0.998

*μ *= 0.004, *B *Optimized	*k *= 0.0210 ± 0.0010	88.6
	*a *= 0.812 ± 0.043	*R*^2 ^= 0.981

*μ *= 0.001, *B *Adapting	*k *= 0.0205 ± 0.0006	94.7
	*a *= 1.065 ± 0.048	*R*^2 ^= 0.988

*μ *= 0.004, *B *Adapting	*k *= 0.0210 ± 0.0011	94.6
	*a *= 0.960 ± 0.065	*R*^2 ^= 0.971

*μ *= 0.001, *D *Optimized	*k *= 0.0212 ± 0.0010	62.0
	*a *= 0.393 ± 0.015	*R*^2 ^= 0.987

*μ *= 0.004, *D *Optimized	*k *= 0.0233 ± 0.0016	70.2
	*a *= 0.446 ± 0.027	*R*^2 ^= 0.967

*μ *= 0.001, *D *Adapting	*k *= 0.0216 ± 0.0011	71.0
	*a *= 0.475 ± 0.020	*R*^2 ^= 0.983

*μ *= 0.004, *D *Adapting	*k *= 0.198 ± 0.0015	80.1
	*a *= 0.586 ± 0.036	*R*^2 ^= 0.967

The Pareto probability distribution function fits the numerically obtained distributions of small effects in all situations studied. Here we show the parameters yielded by the least-squares fit, the *R*-squared value, and the fraction (in percent) of mutations which affect fitness up to 22% (small effect).

**Figure 9:**
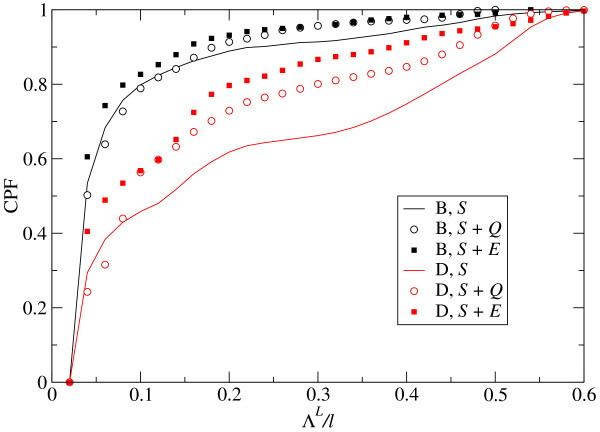
**Cumulative distributions of fitness effects for three different fitness landscapes**. Comparison of the cumulative probability distributions (CPF) of beneficial (*B*-curves) and deleterious (*D*-curves) effects of mutations in fitness for different fitness landscapes: *S *signals selection on structure; *S *+ *Q *represents selection on structure and sequence; *S *+ *E *stands for selection on structure and energy of the folded state. The mutation rate is *μ *= 0.003 in all cases and all populations are optimized: they have attained mutation-selection equilibrium.

Our analysis of optimized populations in different fitness landscapes agrees with the functional behaviour just described. Changes in the fitness landscape modify the distribution of effects on fitness, though only quantitatively. The three landscapes studied are compared in Figure 9, where we show the cumulative probability function *Q*(*x *≤ Λ) for beneficial (*B*) and deleterious (*D*) changes in fitness and the three landscapes analysed. The visible similarity between the functional form in either case is supported by the quantitative analysis of the distribution of small effects, as summarized in Table 4. Comparison with Table 3 reveals a universal statistical behaviour of the functional form of *Q*(*x *≤ Λ) for the landscapes and populations studied.

**Table 4:** Comparison of Pareto fits to the distribution of small effects on fitness for three different fitness landscapes

	Pareto[*k, a*]	% of mutations
*B*	*k *= 0.0197 ± 0.0003	89.9
*S*	*a *= 0.993 ± 0.020	*R*^2 ^= 0.997

*B*	*k *= 0.0200 ± 0.0003	92.2
*S *+ *Q*	*a *= 0.976 ± 0.017	*R*^2 ^= 0.998

*B*	*k *= 0.0198 ± 0.0004	94.2
*S *+ *E*	*a *= 1.170 ± 0.034	*R*^2 ^= 0.995

*D*	*k *= 0.0190 ± 0.0010	63.5
*S*	*a *= 0.391 ± 0.016	*R*^2 ^= 0.984

*D*	*k *= 0.0219 ± 0.0016	75.2
*S *+ *Q*	*a *= 0.529 ± 0.034	*R*^2 ^= 0.963

*D*	*k *= 0.0197 ± 0.0014	81.0
*S *+ *E*	*a *= 0.596 ± 0.036	*R*^2 ^= 0.965

The Pareto probability distribution function fits the numerically obtained distributions of small effects well when selection of a specific 10 nt sequence or selection of low-energy folds occurs simultaneously to selection of a target secondary structure. Results shown as in Table 3. *S *indicates selection solely on structure (according to the definition given in Eq. (1)); *S *+ *Q *stands for populations with selection on structure and sequence (definition given in Eq. (8)); *S *+ *E *represents populations with selection on structure and energy (following Eq. (9)). Distributions are measured for populations optimized at a value of the mutation rate *μ *= 0.003.

#### Average effect of mutation rate on phenotype

In this section, we compare by means of explicit examples the overall effect of mutations, as obtained in our simulations with RNA sequences, with some measures performed in natural systems. Due to the important differences between populations, environments, and fitness definitions used in different works, the effect of mutations in those different systems should be compared with due care. Nevertheless, the values obtained might give clues about the degree of optimization of the population and about the likeliness that fitness is improved. In the following, the numerical results obtained in our simulations correspond to populations evolving on the *S*-landscape.

Let us illustrate, with an example, the overall relative fractions of beneficial, deleterious, and neutral mutations affecting optimized and adapting populations. Take the mutation rate *μ *= 0.001. As described, there is a fraction *P*(0) = 0.951 of replication events without mutations and an amount 1 - *P*(0) - *P*(1) = 1.209 *× *10^-3 ^of daughter sequences incorporating two or more mutations. Hence, in most cases where mutations occur, the replicated sequence is hit by a single mutation (*P*(1) = 0.047). Let us consider only those cases, where a mutation has been incorporated. Our simulations indicate that, at equilibrium, 0.59% of such mutations have beneficial effects, while 46.34% diminish fitness. A large amount of mutations is neutral: 53.07%. Adapting populations suffer a similar amount of neutral mutations, 54.33% at the same mutation rate *μ*, but they substantially differ in the amount of mutations with an effect on fitness. Deleterious mutations represent 38.51%, and beneficial mutations increase to 7.16%. It would be difficult to evaluate the effect of single mutations at higher rates of *μ*. For example, if the average number of mutations per replication event is one per genome (*μ *= 0.02 in our case), over 26% of the daughter genomes incorporate two or more mutations, and epistatic effects should not be discarded. This might entail a difficulty when translating the results of controlled experiments to natural situations involving fast-mutating replicators.

Empirical results concerning the effect of mutations on fitness have been obtained in different contexts and for different model systems. Single nucleotide substitutions in vesicular stomatitis virus yielded complex distributions of fitness effects, with the functional form of those corresponding to beneficial mutations (Γ-distribution) differing from that of deleterious mutations (a Lognormal distribution), and both depending on how mutations were acquired [18]. The overall probability of a deleterious mutation (conditional on a single mutation having taken place) was above 35%, and the probability of beneficial mutations was around 9%. Such a large amount of beneficial mutations suggests that the population was not yet optimized with respect to the fitness trait measured in laboratory assays.

## Conclusions

We have quantified the effect of mutations on fitness for populations of RNA sequences in different situations. Beneficial or compensatory mutations are systematically less probable than deleterious ones, the ratio between both types being strongly dependent on the degree of optimization of the population. Once the error threshold is crossed (at high error rates), optimized and adapted populations behave similarly. Selection coefficients are almost constant, regardless of the degree of adaptation of the population. They are only slightly lower in adapting populations than in optimized populations evolved at the same mutation rate, indicating that adaptation takes place through an increase in the fraction of beneficial mutations, preferably of those with small effect in fitness.

Efforts to quantify the distribution of positive fitness effects rely on the interest to understand the dynamics of the adaptive process. Mutations of small effect are difficult to detect and quantify, so both theory and experiment have mainly addressed the tail of the distribution, where mutations with a large positive effect on fitness sit. Extreme value theory predicted an exponential shape for large effects [21]. However, available data for beneficial mutations in the phage Φ6 rejects the exponential shape and points to a right-truncated distribution [20]. As yet, the distribution of small beneficial effects has not been empirically evaluated. Our numerical results indicate that the whole distribution is compatible with an algebraic decay for small effects up to values around a 25% change in fitness, explaining however up to 90% of mutations. The shape of the distribution of fitness effects is similar for beneficial and deleterious effects, and both can be satisfactorily fit by a Pareto probability distribution.

The results here presented could be used to design more realistic evolutionary models where an explicit representation of the microscopic mutation rate is not feasible. These suggest using phenotypic mutation fractions that increase algebraically with the genomic mutation rate *μ*. In a first approximation, and in the absence of additional evidence, it would be advisable to maintain constant selection coefficients. As a consequence, at least in the system that we have studied, fitness equilibria are reached and maintained mainly through compensatory epistasis, and not through a variation in the average selective coefficients with fitness. This result is in good agreement with recent measurements of the same effects in populations of the bacteriophage ΦX174, where, moreover, the distributions of beneficial and deleterious fitness effects follow a single functional form [6], as obtained here.

The restrictions imposed by the simultaneous selection of more than one phenotypic trait is an important subject worthy of additional analysis. Landscapes such as those introduced here could be a first step towards the quantification of constraints in evolution and adaptation of complex populations. Despite remarkable differences among the situations investigated, the distributions of effects on fitness are best explained by a unique functional form (at least for small effects) irrespectively of the state of adaptation of the population (whether optimized or adapting) and of the fitness landscape on which the population evolves (*S, S *+ *Q*, or *S *+ *E*). One of our future objectives is to deepen the study of the universality of these and similar models, analyzing whether populations differing in their fitness values across environments, evolving towards different target structures, or incorporating a larger number of realistic phenotypic traits, still adapt through an increase in the amount of beneficial mutations with small effects on fitness, and whether the functional form of the distribution of fitness effects agrees with those obtained here.

## Methods

Simulations have been carried out at the Itanium II cluster of INTA (Instituto Nacional de Técnica Aeroespacial, Spain). For random number generation, we relied on the Mersenne Twister and Ziff's GFSR4 algorithms as provided by GNU Scientific Library (GSL), Version 1.7 [39]. For secondary structure folding (minimum free energy) and calculation of base-pair and Hamming distances, we use the Vienna RNA package [32], version 1.5, with the current standard parameter set. Nonlinear regressions to probability distribution functions were performed with the Statistics packages of Mathematica 5.2.

## Authors' contributions

MS, EL, and SCM conceived and designed the research. MS performed the simulations. MS, EL, and SCM analyzed the data. EL and SCM performed the statistical analysis. SCM wrote the paper. All authors read and approved the final manuscript.
